# The impact of the COVID-19 pandemic-related quarantine on female sexual behavior: a cross-sectional study in China

**DOI:** 10.1038/s41598-022-23974-4

**Published:** 2022-11-12

**Authors:** Guangyong Li, Puguang Yu, Fen Zhang, Yanlong Xu, Gaiyan Zhou, Xuekang Zhan, Yu Gao, Xiaoli Du, Hetao Liu, Rui He

**Affiliations:** 1 The General Hospital of Ningxia Medical University, Ningxia Medical University, Yinchuan, 750000 China; 2 Key Laboratory of Fertility Preservation and Maintenance of Ministry of Education, Ningxia Medical University, Yinchuan, 750004 China; 3 Jinshan Hospital of Fudan University, Fudan University, Shanghai, 201500 China; 4 The Affiliated Brain Hospital of Nanjing Medical University, Nanjing Medical University, Nanjing, China

**Keywords:** Endocrinology, Health care, Medical research, Risk factors

## Abstract

To investigate the impact and factors of home quarantine life on women’s sexual lives and behaviors in different areas of China and analyze the prevalence of female sexual dysfunction (FSD) during the COVID-19 pandemic. We surveyed adult women who had a regular sexual life (including regular masturbation) and had been isolated at home for at least one month during the COVID-19 outbreak using online questionnaires. This survey recovered 678 complete questionnaires after screening. According to the findings, the overall score of the Female Sexual Function Inventory (FSFI) during the pandemic was 21.98 ± 6.38, the frequency of FSD was 61.9%, and the frequencies of FSD in Shanghai, Nanjing, and Ningxia were 60.6%, 75.2%, and 52.2%, respectively. The frequency of FSFI scores and other specific items (Desire, Arousal, Lubrication, Orgasm, Satisfaction, and Pain) varied significantly across the three regions (*P* < 0.05). The overall frequency of FSD in the masturbation population was 34.4%, which was lower than the frequency of FSD in women having paired sexual intercourse (60.1%) (*p* < 0.05). Further analysis revealed that the occurrence of FSD during the pandemic was related to different age stages, menopause, mode of delivery, level of anxiety and depression, and sexual lifestyles. The COVID-19 pandemic has had a great impact on people’s spiritual and sexual lives, which are caused by multiple different variables related to both the individual and the environment. We should emphasize the importance of sexual health in epidemics, and having a harmonious and stable sex life will help us survive the boring life of isolation.

## Introduction

It has been more than 2 years since the global outbreak of the COVID-19 pandemic in March 2020. Compared with the previous freedom in our social lives, pandemic-related quarantines (isolation at home) has not only affected our activities but also mostly influences mental health, and sexual health is one of the most affected and overlooked issues, especially women’s sexual health^1^. Female sexual dysfunction (FSD) is a highly incidence and poorly understood condition characterized of unwanted, unremitting sensations of genital arousal, and previous research have found it to be strongly linked to menopause^2^, interpersonal relationships^3^, and cultural^4^ and so on. Particularly, the prevalence of sexual problems is different from country to country concerning culture. In countries like Iran, female genital mutilation is practiced in parts of underage women, and talking about sexuality or sexual research in public can have disastrous consequences^5^, which makes the incidence of FSD difficult to assess in these countries. In the Chinese, people’s thinking is relatively conservative and the topic of sexuality is gradually being talked about, but this is just the beginning. Therefore, compared to countries with earlier sexual openness, we guess that the incidence of FSD in the Chinese population is much higher than that in developed countries under the influence of multiculturalism^6^.

The impact of the epidemic on women’s sexual behavior is obvious. The study of Fuchs et al.^7^ discovered that after social quarantine, the overall Female Sexual Function Index (FSFI) score and other domain scores all decreased, indicating that women’s sexual health is facing great challenges during the pandemic. In fact, sexual health is an important part of women’s physical and mental health^8^; it not only affects women’s normal lives, fertility and social interactions but also affects and threatens their male partners’ sexual health^9^.At present, the situation in China regarding epidemic prevention remains dire, and the government requires residents who live in high-risk areas to be quarantined at home for 4 weeks to prevent a large-scale outbreak, which has had an unprecedented impact on people’s routine lives. Previous related studies have shown that pandemic-related quarantines may have different effects on women’s sexual health. This has been reported in Italy^10^, Turkey^11^ and other countries^12^ but has rarely been reported in China.

Therefore, this investigation aimed to analyze the impact of the pandemic on women’s sexual lives and sexual behaviors in three regions of China (i.e., Shanghai, Nanjing, and Ningxia) and to understand the current situation of women’s sexual lives and the prevalence of FSD during the COVID-19 pandemic-related quarantine. Furthermore, we anticipate that our research can provide guidance and a reference for women’s sexual health during the pandemic.

## Results

### Characteristics of the study population

Since the questionnaires in this investigation were granted and collected through the internet, it was not possible to calculate the exact recovery rate. We eliminated the clearly unqualified questionnaires (completed too fast and/or the same option was selected for all questions). Ultimately, 678 questionnaires were received; among them, 221 were from Shanghai, 206 were from Nanjing, and 251 were from Ningxia. The characteristics of the study population are given in Table 1. Among all respondents, the age distribution was mainly concentrated in the 18–40 years age group (81.3%); fortunately, these women were in the sexually active period. The majority of respondents in the survey were married (65.2%), and unmarried (including divorced or widowed) women accounted for the minority (34.8%). The most representative education level was a bachelor’s degree (67.4%). In terms of menstruation, almost 9 out of 10 participants had a normal and regular menstrual cycle (87.9%). Among all participants, only 12.1% had experienced menopause. Regarding childbirth, 267 (39.3%) participants had no history of parturition; regarding delivery methods, 50.1% had a vaginal delivery, 14.9% had a caesarean section, and 35% had no delivery.

**Table 1:** The characteristics of the study population (n = 678).

Items	Total (n = 678)	Shanghai (221)	Nanjing (206)	Ningxia (251)
**Age (years), n (%)**				
18–25	159 (23.5)	46 (20.8)	32 (15.5)	81 (32.3)
26–30	204 (30.1)	43 (19.5)	90 (43.7)	71 (28.3)
31–40	188 (27.7)	82 (37.1)	63 (30.6)	43 (17.1)
41–50	100 (14.7)	39 (17.6)	21 (10.2)	40 (15.9)
51–60	22 (3.2)	10 (4.5)	0	12 (4.8)
> 60	5 (0.7)	1 (0.5)	0	4 (1.6)
**Job, n (%)**				
Student	119 (17.6)	32 (14.5)	26 (12.6)	61 (24.3)
Production and sales staff	221 (32.6)	59 (26.7)	89 (43.2)	73 (29.1)
Administrative staff	127 (18.7)	66 (29.9)	32 (15.5)	29 (11.6)
Service personnel	79 (11.7)	19 (8.6)	7 (3.3)	53 (21.1)
Professional skilled worker	132 (19.5)	45 (20.4)	52 (25.2)	35 (13.9)
**Educational level, n (%)**				
Primary school	14 (2.1)	0	2 (1.0)	12 (4.8)
Junior high school	54 (8.0)	8 (3.6)	11 (5.3)	35 (14.0)
High school or secondary school	153 (22.6)	29 (13.1)	21 (10.2)	103 (41.0)
College and above	457 (67.4)	184 (83.3)	172 (83.5)	101 (40.2)
**Marital status, n (%)**				
Unmarried	196 (28.9)	88 (40.0)	51 (24.8)	57 (22.7)
Married	442 (65.2)	124 (56.1)	141 (68.4)	177 (70.5)
Divorced or widowed	40 (5.9)	9 (4.1)	14 (6.8)	17 (6.8)
**Menstruation, n (%)**				
Yes	82 (12.1)	18 (8.1)	26 (12.6)	38 (15.1)
No	596 (87.9)	203 (91.9)	180 (87.4)	213 (84.9)
**Reproductive history, n (%)**				
Not pregnancies or births	267 (39.3)	76 (28.5)	71 (34.1)	100 (37.5)
1 birth	278 (41.0)	79 (28.4)	69 (35.6)	68 (24.5)
2 births	106 (15.6)	61 (57.5)	45 (42.5)	52 (49.1)
3 births and above	27 (4.0)	5 (18.5)	21 (77.8)	31 (87.1)
**Mode of delivery, n (%)**				
No births	237 (35.0)	71 (32.1)	86 (41.7)	80 (31.9)
Vaginal delivery	340 (50.1)	113 (51.1)	72 (35.0)	155 (61.8)
Cesarean section	101 (14.9)	37 (16.7)	48 (23.3)	16 (6.4)

### The impact of the COVID-19 pandemic on sexual behavior

In general, the forced pandemic-related quarantine had a great impact on women’s mental health and sexual lives. As presented in Table 2, 63.6% of the respondents experienced anxiety during this period, and 58.8% suffered from depression. Regarding sexual life, 68.4% of the respondents reported that the quarantine at home had a great (more than half of the time) impact on their sexual life, 29.4% said that their sexual desire was lower than before, 32.1% indicated that their sexual desire had increased, and only a small number (38.5%) said that their sexual desire had not been affected by the pandemic. We found that the impact of the pandemic on sex was bidirectional, with both positive and negative effects. By analyzing the factors, we found that the increase in sexual desire was mainly because of spending sufficient with their sexual partners during home isolation (35.1%), and the pressure from life and work was less than before the pandemic (38.9%). Other reasons include sufficient recuperation that produced plenty of energy. In addition, the excellent performance of sexual partners was also a relatively important reason. For participants with lower desire, the substantial psychological pressure (43.4%) and the heavy study (housework) tasks (32.9%) were the main reasons for this phenomenon, and other reasons included gradually losing interest in having the same sex partners for a long time (6.2%) and the reasons for the sexual partners themselves (18.6%). Apart from this, to our surprise, 53.1% of the women used masturbation to meet their sexual needs, and 29.1% used sex products during the quarantine period. In this study, we also found that 13.7% of the respondents indicated that they had little knowledge about sex, with almost no knowledge, 74.2% had a small amount of knowledge about sex, and only 12.1% said their knowledge was very sufficient. Among the respondents, 71.7% believed that their knowledge of sexuality was lacking and wanted further study, and only 28.2% said they had sufficient sexual knowledge and no longer needed to study.

**Table 2:** The impact of the COVID-19 pandemic on sexual behavior (n = 678).

Item	Total (678)	Shanghai (221)	Nanjing (206)	Ningxia (251)
**The extent of the impact of home isolation on sexual life, n%**				
< 50%	214 (31.6)	67 (30.3)	81 (39.3)	66 (26.3)
> 50%	464 (68.4)	154 (69.7)	125 (60.7)	185 (73.7)
**The extent of the impact of home isolation on sexual behavior, n%**				
No impact	199 (29.4)	63 (28.5)	35 (17.0)	101 (40.2)
Less than before	261 (38.5)	64 (29.0)	89 (43.2)	108 (43.0)
More than before	218 (32.1)	94 (52.5)	82 (39.8)	42 (16.7)
**Reasons for increased sexual behavior during quarantine at home, n%**				
Spending a lot of time together	238 (35.1)	75 (34.0)	62 (30.1)	101 (40.2)
Less mental stress	264 (38.9)	76 (34.4)	80 (38.8)	108 (43.0)
Increased energy	105 (15.9)	46 (20.8)	32 (15.5)	27 (10.8)
Sexual partner behaves well	71 (10.5)	24 (10.9)	32 (15.5)	15 (6.0)
**Reasons for decreased sexual behavior during quarantine at home, n%**				
Great pressure about the pandemic	294 (43.4)	84 (38.0)	72 (35.0)	138 (55.0)
Heavy study/housework/work tasks	201 (29.6)	73 (33.0)	68 (33.0)	60 (24.0)
Losing interest in having the same sex partner	88 (13.0)	22 (10.0)	35 (17.0)	31 (12.4)
Sexual partner’s own factors	95 (14.0)	42 (19.0)	31 (15.0)	22 (8.8)
**Style of sexuality, n%**				
Masturbation	163 (24.0)	64 (29.0)	40 (19.4)	59 (23.5)
Sex products	197 (29.1)	56 (25.3)	58 (28.2)	83 (33.1)
Paired sexual intercourse	318 (46.9)	101 (45.7)	108 (52.4)	109 (43.4)
**Sexual knowledge, n%**				
Hardly any	93 (13.7)	19 (20.4)	26 (28.0)	48 (51.6)
< 50%, Need further learning	433 (74.2)	159 (31.6)	103 (20.5)	171 (47.9)
> 50%, No need to learn	152 (12.1)	43 (42.7)	77 (25.6)	32 (31.7)
**Do you want to learn more about sexual knowledge and skills? n%**				
Yes	486 (71.7)	163 (73.8)	139 (67.5)	184 (73.3)
No	192 (28.3)	58 (26.2)	67 (32.5)	67 (26.7)
**Anxiety occurred during the pandemic, n%**				
Yes	431 (63.6)	161 (72.9)	129 (62.6)	141 (56.2)
No	247 (36.4)	60 (27.1)	77 (37.4)	110 (43.8)
**Depression occurred during the pandemic, n%**				
Yes	492 (72.6)	173 (78.3)	151 (73.3)	168 (67.0)
No	186 (27.4)	48 (21.7)	55 (26.7)	83 (33.0)

### The mean scores of the Female Sexual Function Index and the prevalence of FSD in different regions

The mean scores of the Female Sexual Function Index (FSFI) and other specific items (desire, arousal, lubrication, orgasm, satisfaction, and pain) are shown in Table 3. Analysis of the scores of female sexual function, measured with the FSFI, showed that, as expected, there were differences in the FSFI total score and each dimension score in the three regions (*P* < 0.05), and the Ningxia region scored lower than the other two regions (FSFI: 15.24 ± 4.58 vs. 23.38 ± 1.30 and 28.33 ± 2.37, respectively, and other items are shown in Table 3). Furthermore, we conducted statistical analysis on the frequency of FSD and other items in the three regions, which fortunately were consistent with the above scores: we found that there were statistically significant differences in the frequency of various types of sexual dysfunction in the three regions (*P* < 0.05). The overall frequency of FSD was 61.9%, and the frequencies in Shanghai, Nanjing, and Ningxia were 60.6%, 75.2%, and 52.2%, respectively (Table 4). Among the different types of sexual dysfunction, low sexual satisfaction was the highest (43.8%), sexual arousal difficulty was the second highest (35.1%), and the frequency of pain during intercourse was the lowest (21.8%). The frequencies of various types of sexual dysfunction in the three different regions are shown in Table 4.

**Table 3:** The mean scores of the Female Sexual Function Index (FSFI) (mean ± SD).

	Total	Shanghai	Nanjing	Ningxia	*F*	*P*
Desire	3.58 ± 1.22	4.66 ± 0.74	3.83 ± 50.6	2.44 ± 0.60	482.20	**0.032**
Arousal	3.65 ± 1.82	4.77 ± 0.56	3.85 ± 0.49	2.50 ± 0.91	638.47	**< 0.001**
Lubrication	3.76 ± 1.21	4.82 ± 0.59	4.00 ± 0.61	2.63 ± 1.01	473.38	**< 0.001**
Orgasm	3.63 ± 1.20	4.74 ± 0.61	3.88 ± 0.56	2.45 ± 0.87	638.96	**0.011**
Satisfaction	3.80 ± 1.21	4.85 ± 0.59	4.08 ± 0.59	2.65 ± 0.99	509.66	**0.029**
Pain	3.56 ± 1.15	4.50 ± 0.86	3.75 ± 0.63	2.58 ± 0.93	328.63	**< 0.001**
FSFI	21.98 ± 6.38	28.33 ± 2.37	23.38 ± 1.30	15.24 ± 4.58	1024.85	**< 0.001**

FSFI, Female Sexual Function Index.

Significance values are in bold.

**Table 4:** Prevalence of different types of sexual dysfunction in different regions (n%).

	Total (678)	Shanghai (221)	Nanjing (206)	Ningxia (251)	F	*P*
Desire (< 3.6)	226 (33.3)	97 (42.1)	41 (19.9)	88 (35.1)	141.25	**< 0.001**
Arousal (< 3.6)	238 (35.1)	65 (29.4)	82 (39.8)	91 (36.3)	184.37	**0.031**
Lubrication (< 3.9)	171 (25.2)	44 (19.9)	59 (28.6)	68 (27.1)	103.24	**< 0.001**
Orgasm (< 4.0)	188 (27.7)	56 (25.3)	61 (29.6)	71 (28.3)	99.23	**< 0.001**
Satisfaction (< 4.4)	297 (43.8)	104 (47.1)	92 (44.7)	101 (40.2)	83.24	**< 0.001**
Pain (< 4.5)	148 (21.8)	51 (23.1)	33 (16.1)	64 (25.5)	142.24	**0.026**
FSD (FSFI score < 26.55)	420 (61.9)	134 (60.6)	155 (75.2)	131 (52.2)	95.13	**< 0.001**

FSD, female sexual dysfunction; FSFI, Female Sexual Function Index.

Significance values are in bold.

### Correlation analysis of FSD

To assess the risk of FSD, correlation analysis was performed to assess the relationships between variables. After the cause analysis, as shown in Table 5, it was found that the occurrence of FSD was mainly related to marital status (β = − 0.633, *p* = 0.015), whether menopause had occurred (β = − 0.086, *p* = 0.024), mode of delivery (β = − 0.087, *p* = 0.026), childbirth history (β = − 0.056, *p* = 0.011), degree of depression (β = − 0.942, *p* = 0.012) and anxiety (β = − 0.342, *p* = 0.038). Regrettably, job, age, education level, and childbirth history were not significantly correlated.

**Table 5:** Correlation analysis of FSD occurrence by logistic regression.

Variable	β	SE	χ^2^	*P*	OR (95% CI)
Job	0.282	0.082	11.810	0.396	1.325 (1.12, 1.56)
Age	− 0.001	0.129	0.000	0.996	0.999 (0.77, 1.29)
Marital status (unmarried)	− 0.633	0.261	5.900	**0.015**	0.531 (0.31, 0.89)
Education level	− 0.215	0.154	1.944	0.163	0.806 (0.59, 1.09)
Whether menopause has occurred	− 0.086	0.328	0.069	**0.024**	1.090 (0.57, 2.04)
Childbirth history	− 0.056	0.140	0.160	**0.011**	1.057 (0.80, 1.39)
Mode of delivery	− 0.087	0.206	0.180	**0.026**	0.916 (0.61, 1.32)
Depression	− 0.942	0.513	3.377	**0.012**	0.361 (0.21, 0.76)
Anxiety	− 0.342	0.438	4.867	**0.038**	0.534 (0.32, 0.81)

CI, confidence interval; OR, odds ratio; β, standardized coefficients; ${\text{S}}_{{{\overline{\text{X}}}}}$, standard deviation of the population mean.

Significance values are in bold.

### The prevalence of FSD between masturbation and paired sexual intercourse in different regions and correlation analysis

We conducted a separate analysis of this group of women who used masturbation to satisfy their sexual needs, so we divided the participants into a masturbation group and a paired sexual intercourse group. A statistically significant difference was observed regarding the prevalence of different types of sexual dysfunction between the 2 study groups in the three regions. We found that the frequency of desire was lower in the masturbation group than in the paired sexual intercourse group (Total: 36.9 vs. 30.2; Shanghai: 47.1 vs. 42.2; Nanjing: 27.6 vs. 26.9; Ningxia: 34.4 vs. 51.4, respectively). Conversely, apart from this, for other items (including arousal, lubrication, orgasm, satisfaction, pain and FSFI score), the masturbation group scored lower than the paired sexual intercourse group (*p* < 0.05) (shown in Table 6). Through further correlation analysis, we found similar and different risks of FSD between the masturbation and paired sexual intercourse groups: age, educational level, depression and anxiety were the same factors influencing the occurrence of FSD. In addition, in participants in the masturbation group, the style of sexuality and sexual knowledge were important factors influencing FSD. Similarly, reproductive history, mode of delivery and educational level were independent risk factors in the paired sexual intercourse group (all *P* < 0.05) (Table 7).

**Table 6:** Prevalence of different types of sexual dysfunction related to masturbation and paired sexual intercourse in different regions (n%).

Item	Total (678)		Shanghai (221)		Nanjing (206)		Ningxia (251)		*F*	*P*
	Masturbation (360)	Paired sexual (318)	Masturbation (119)	paired sexual (102)	Masturbation (87)	Paired sexual (119)	Masturbation (154)	Paired sexual (97)		
Desire (< 3.6)	133 (36.9)	96 (30.2)	56 (47.1)	43 (42.2)	24 (27.6)	32 (26.9)	53 (34.4)	50 (51.4)	71.25	**0.011**
Arousal (< 3.6)	99 (27.5)	114 (35.8)	39 (32.8)	33 (32.4)	19 (21.8)	22 (18.5)	41 (26.6)	62 (63.9)	66.24	**0.045**
Lubrication (< 3.9)	129 (35.8)	186 (58.5)	31 (26.1)	52 (51.0)	59 (25.3)	71 (59.6)	39 (25.3)	34 (35.1)	112.14	**< 0.001**
Orgasm (< 4.0)	70 (19.4)	144 (45.3)	19 (20.0)	58 (56.9)	21 (24.1)	84 (70.6)	30 (19.5)	61 (62.9)	38.92	**< 0.001**
Satisfaction (< 4.4)	112 (31.1)	166 (52.2)	51 (42.9)	77 (75.5)	17 (19.5)	65 (54.6)	44 (28.6)	59 (60.8)	63.27	**0.029**
Pain (< 4.5)	75 (20.8)	143 (45.0)	18 (15.1)	41 (40.2)	9 (10.3)	35 (29.4)	48 (31.2)	56 (57.7)	66.78	**< 0.001**
FSD (FSFI < 26.55)	124 (34.4)	191 (60.1)	34 (28.6)	66 (64.7)	39 (44.8)	86 (72.3)	51 (33.1)	71 (73.2)	295.44	**< 0.001**

FSFI, Female Sexual Function Index.

Significance values are in bold.

**Table 7:** Correlation analysis of FSD occurrence related to masturbation and paired sexual intercourse by logistic regression.

Masturbation							Paired sexual intercourse						
Variable		β	SE	χ^2^	*p*	OR (95% CI)	Variable		β	SE	χ^2^	*p*	OR (95% CI)
Age		− 0.290	0.121	5.788	**0.016**	0.748 (0.59, 0.95)	Age		− 0.163	0.094	2.919	0.031	1.136 (0.13, 0.79)
Depression	Yes	− 0.479	0.713	1.335	**0.022**	0.434 (0.33, 1.87)	Childbirth history	≥ 1	− 0.277	0.466	1.467	0.012	0.565 (0.43, 0.68)
Anxiety	Yes	− 0.913	0.662	0.972	**0.031**	1.626 (0.13, 1.24)	Mode of delivery	Cesarean section	− 0.336	0.412	1.072	0.031	0.224 (0.96, 1.13)
Style of sexuality	masturbation	− 0.413	0.135	9.277	**0.002**	0.662 (0.50, 0.86)	Educational level		− 0.833	0.220	3.188	0.022	0.551 (0.31, 0.77)
Educational level		− 0.147	0.192	0.589	**0.043**	0.863 (0.59, 1.26)	Anxiety	Yes	− 0.099	0.219	0.997	0.003	0.461 (0.22, 0.96)
Sexual Knowledge	> 50%, need further learning	− 0.663	0.112	1.232	**0.009**	1.681 (0.31, 2.12)	Depression	Yes	− 0.535	0.339	0.675	0.001	0.868 (0.44, 1.22)

Significance values are in bold.

## Discussion

During the COVID-19 pandemic and after social isolation, several aspects of daily life dramatically changed. This has had an unprecedented impact on our spiritual lives, including our sexual health, which we often overlook. With the increase in pressure and the acceleration of life’s pace, there are many potential threats to women’s sexual health, which not only cause women physical distress^13^ but also put a substantial psychological burden on them, even threatening the sexual function of their male partners^9^. To date, few studies from China on female sexual dysfunction have been reported during the COVID-19 pandemic; in addition, no study has evaluated women who use masturbation to meet their sexual needs as an independent population to study the incidence of FSD and analyze risk factors.

In this cross-sectional study, we selected the three most representative regions in terms of economy, culture, and educational level in China (i.e., Shanghai/Nanjing/Ningxia) for investigation, which can best represent the characteristics of the Chinese population in this regard. Also as we predicted, there was a significant difference in the frequency among the three regions (*p* < 0.05), this was because there were regional differences in the occurrence of FSD, which was closely related to the economic and education levels of the residents^14^. We found that the overall frequency of FSD during the new coronavirus pandemic was 61.9%, which was much higher than that in China before the pandemic (29.7%)^15^, this is consistent with the conclusions of Italian study during the pandemic that the negative impact of the COVID-19 epidemic period on sexual function^16^. These data suggest that living in isolation indeed has a great impact on women’s sexual behaviors, which is worthy of further attention to this issue. In addition, we also found that in Shanghai and Nanjing, the scores or frequencies in terms of different types of sexual dysfunction were significantly higher than those in Ningxia (*P* < 0.05) because Shanghai and Nanjing are developed regions in China, including economic and educational^17^. Furthermore, we analyzed the factors that may affect the occurrence of FSD and found that marital status, menopause, mode of delivery, and the degree of depression and anxiety were mainly related to FSD (*P* < 0.05), which was similar to the results of previous studies^18^; that is, psychological factors are an important cause of FSD. In addition, there are some other factors related to organic lesions of the external genitalia, such as mechanical damage to the pelvic floor^19^ and sexual organs caused by prolonged labor during childbirth^20^, and the sexual organs, such as vaginal loss of nutrition and atrophy caused by the decline in sex hormones caused by menopause^21^, which causes damage to the normal structure of the vagina, in turn causing vaginal dysfunction.

The COVID-19 pandemic has had a substantial impact on our original family models and relationships^22,23^. This is primarily due to the panic caused by the pandemic and the fear of being infected. In our survey, overall, home isolation during the pandemic had an unprecedented impact on people’s lives, and almost more than half of the population reported experiencing anxiety (63.6%) and depression (58.8%) during the pandemic; however, a stressful lifestyle was a factor affecting female libido and the frequency of sexual intercourse^24^. Deng et al*.*^25^ analyzed 31 studies and found that the overall prevalence of depression was 45% during the pandemic, the overall prevalence of anxiety was 47%, and 34% of the population suffered from sleep disturbances. This indicates that we should all be concerned about mental health during the epidemic,, including policy-makers and psychologists.

Nevertheless, under the tense atmosphere of the COVID-19 pandemic, people’s sexual lifestyles have inevitably changed. Through the survey, we found that only a small number (38.5%) of people said that home isolation had no effect on their sexual desire, while most of the respondents (68.4%) said it had a great impact on their sexual life. Among the affected people, 29.4% indicated that their sexual desire was lower than before the pandemic, and the reasons for the decline included the substantial psychological pressure caused by the pandemic, the heavy study and housework tasks during the quarantine period, having the same sex partner for a long time and sexual partners’ own factors, which was consistent with Cito’s research^10^. A total of 44% of the participants reported a decrease in the number of sexual partners, and approximately 37% of the participants reported a decrease in sexual frequency^26^. A total of 32.1% of the participants indicated that their sexual desire had increased after the pandemic compared with before the pandemic. The main reasons were sufficient close interaction with their sexual partners at home during the quarantine, less pressure from life and work during the quarantine period than before the pandemic, and other reasons including isolation periods leading to high energy and excellent performance by sexual partners. Previous studies have also analyzed the impact of natural and man-made disasters on women’s sexual behaviors.

Prior to the pandemic, most spouses were’ preoccupied with work, people spent very little time getting along and interacting with their partner, and the relationship and emotional communication was significantly decreased. Of course, the number of partnered sexual activities also decreased^27^. When the pandemic began to isolate people at home, it offered them more contact time, which helped them to re-establish intimacy and improve their sexuality^23^. A study investigated the impact of the Wenchuan earthquake on women’s reproductive health and found that after the earthquake, the frequency of sexual intercourse, sexual life satisfaction, and desire for children decreased^28^. Compared with the earthquake, the psychological trauma caused by the pandemic is a moderate and acceptable process, and its impact is not as severe. Therefore, in our study, the number of people with increased libido outnumbered those with decreased libido, indicating that home isolation during the pandemic may promote sexual behavior. However, several similar studies have come to the opposite conclusion: a study on the quality of sexual life of Polish women during the pandemic showed that both sexual dysfunction scores and sexual intercourse frequency were lower than before the pandemic^7^, and another study also examined the impact of the COVID-19 pandemic on female sexuality in Turkish women. The results also showed that the pandemic had a negative effect on sexual life^11^. In the survey, we found that home isolation caused women to feel less exhausted from their hard work, making them more vibrant, which is undoubtedly a more effective method to promote sexual life. However, this is not entirely the case. A total of 29.4% of the participants indicated that long-term home isolation had reduced their sexual desire and sexual conduct, which made them anxious and melancholy. In addition, they were busier than before, having to take care of children, housekeeping, etc., and in addition to having the same sexual partners for a long time, these reasons led to reduced libido. Therefore, during the pandemic, we should properly adjust the law of life and actively intervene in the emotions between husbands and wives as much as possible, assisting them in better overcoming the pandemic and promoting family harmony and stability.

For a long time, due to the influence of traditional culture and religious and incorrect sexual guidance, masturbation has been regarded as undeserved and sinful. This phenomenon is more common in countries with relatively underdeveloped economic and educational levels^29^. Because of this, most previous studies have tended to focus on people who have regular paired sexual intercourse, and few population-based surveys have included measures of masturbation in their studies. However, in real life, masturbation is an important way for people to meet their sexual needs and is an integral part of normal sexual development^30^; more seriously, the pandemic is affecting teens’ physical, emotional and sexual activities, which are ignored^31^. A British study found that 86% of men and 57% of women aged 16–44 years had masturbated regularly in the past year^32^. Therefore, in this study, we classified divorced, widowed or unmarried people who used masturbation to meet their sexual needs for a long time into a single subgroup to observe the impact of the pandemic. We found that among all the respondents, more than half (53.1%) used masturbation to satisfy their sexual needs, and their masturbation methods included self-stimulation (24.0%) and the use of sexual products (29.1%).

Compared with that in the paired sexual intercourse group, we found that the incidence of FSD in the masturbation group was only 28.6%, with desire in the masturbation group being higher than that in the paired sexual intercourse group; conversely, in other items (including arousal, lubrication, orgasm, satisfaction, pain and FSFI scores), the masturbation group scored lower than the paired sexual intercourse group (as shown in Table 6). We hypothesized that compared to masturbation (including the use of sexual products), emotional and attractive sexual partners arouse libido more easily. In contrast, masturbation can reduce discomfort, and women can easily adjust on their own,, which could be because masturbation is more likely to improve the sexual satisfaction of women^33^. Similar studies also found that women who are satisfied with their sex life seem to be more likely to use masturbation as a complement to perfect sex^34^. This was also confirmed in our study: the incidence of FSD was lower in the masturbation group than in the paired sexual intercourse group (*p* < 0.05), and 36.2% of the women who had regular paired sexual intercourse also masturbated, which further shows that masturbation is not only a compensatory behavior but also an important way to pursue a more perfect sexual experience^34^. David L also confirmed that women who combine masturbation with paired sexual intercourse are more likely to experience orgasm and enhance their orgasmic pleasure^35^. In this study, we also found that masturbation and sexual intercourse not only have the same risk factors but also have their own unique risk factors: age, depression and anxiety are the same factors influencing the occurrence of FSD. These factors appear to be present in all patients with FSD^7,36^, which means aging and mental health should be closely considered, which may be an effective way to prevent the occurrence of FSD. In addition, in the masturbation group, the style of sexuality, the type of job and sexual knowledge were important factors of FSD. Without a doubt, women have more sexual knowledge, and they can know how to meet their sexual needs. Similarly, childbirth history, mode of delivery and educational level were independent risk factors in the paired sexual intercourse group.

Sex education and the popularization of sex knowledge are relatively insufficient in developing countries, which may be the greatest potential risk factor of FSD^37^. The present study also demonstrated that among all the respondents, 74.2% of all respondents said they had little sexual knowledge and needed further study, and 13.7% even said that they had no sexual knowledge at all, which means that people’s sexual knowledge is generally lacking. This is a significant contributor to the prevalence of FSD and the quality of sexual life^38^. Among all factors related to FSD, knowledge about sex is probably the most worthy of doctors’ and the general public’s attention, so we should strengthen the publicity and popularization of sexual knowledge in daily life, which may be the most effective way to prevent FSD.

## Conclusion

Our research shows that the COVID-19 pandemic has had a significant impact on people’s spiritual lives and sexual lives. This impact has a negative impact on mental health, but it also has both negative and positive effects on people’s sex lives, which is mainly caused by it being determined by the characteristics of being isolated at home. In addition, we comparatively analyzed the sexual health status and influencing factors of sexual intercourse and masturbation groups during the pandemic, which helped us understand the sexual health of the masturbating population. Finally, we also found that sex knowledge and education are lacking in many women, which may be the root cause of unhealthy sex. We should emphasize the importance of sexual health in epidemics, and having a harmonious and stable sex life will help us survive the boring life of isolation.

## Methods

### Objectives

**Figure 1:**
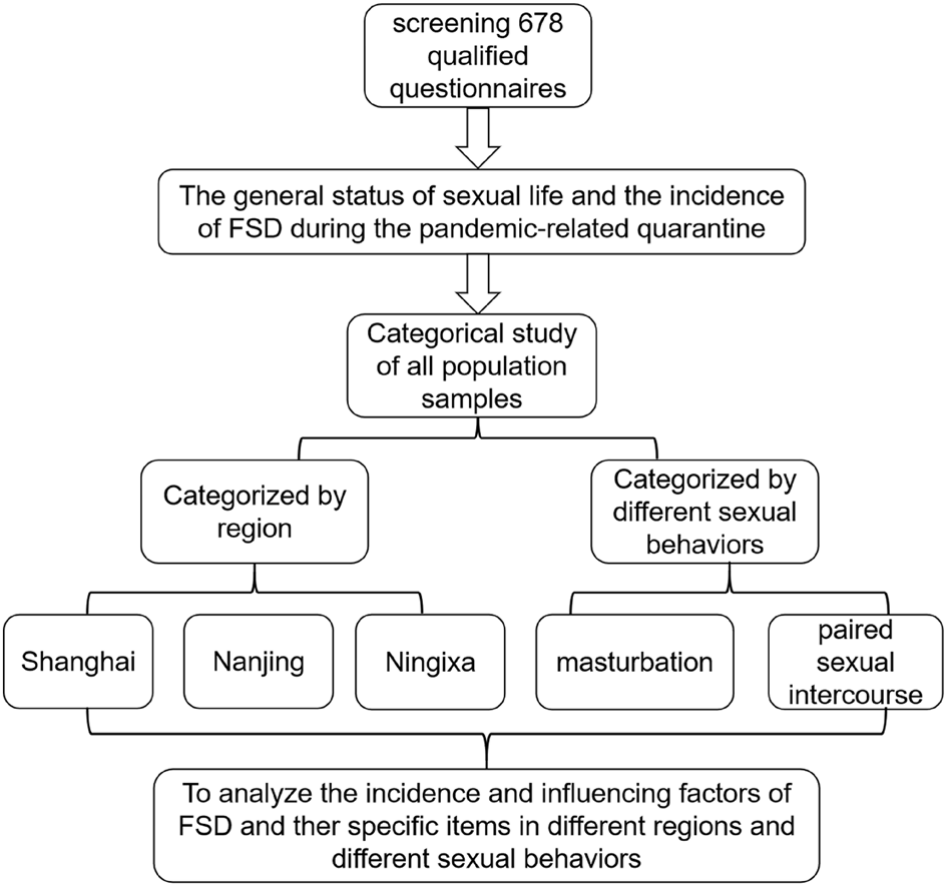
Flowchart for the creation of the process and overview of the investigation. FSD, female sexual dysfunction.

Our entire experimental design and process are shown in Fig. 1. We selected women who were sexually active (including regular masturbation) during home isolation for at least 1 month after the start of the COVID-19 pandemic-related quarantine. The inclusion criteria were as follows: (1) women aged over 18 years; (2) women who lived in the local area for more than 5 years; (3) women with a regular sexual life during the quarantine period, including paired sexual intercourse and masturbation; and (4) women with an appropriate level of education who could understand the content of the questionnaire and voluntarily cooperate with the survey. The exclusion criteria were as follows: (1) women who did not have sexual lives; (2) women who had a mental illness and could not cooperate with the investigation; (3) women who had abstained from sexual intercourse for nearly one month due to pregnancy, breastfeeding, gynecological inflammation, etc. (4) women with a history of long-term psychiatric or hormonal drug treatment; (5) women with gynecological illnesses/conditions affecting their sexual function, such as genital tract malformations and malignant tumors; and (6) women with sexual dysfunction due to spinal cord or cauda equina injury.

The survey was conducted on the basis of voluntary participation. Before the investigation, we fully informed the respondents of the purpose of the study and the details of the questionnaire; the purpose of the study and informed consent was marked prominently in the questionnaire, and informed consent was obtained from all the participants. In addition, this study was approved by the Ethics Committee of the General Hospital of Ningxia Medical University (ethics number: 2022-23), and was performed in accordance with the Declaration of Helsinki.

### Methods

The data were collected from February 2020 to March 2022. To protect the privacy of participants, we distributed electronic questionnaires (https://www.wjx.cn/vj/YOpC6HW.aspx) through social software (Weibo, WeChat, QQ and e-mail), and to ensure the quality of the questionnaire, we set the shortest completion time and other precautions to ensure the authenticity and reliability of the data. The questionnaire consisted of three parts. The first part included basic information, including age, occupation, education level, marital status (menstrual status, marital status, mode of production, number of births), etc. The second part concerned the impact of the pandemic on sexual life, including the degree of impact of isolation due to the pandemic on married life, psychological changes (anxiety, depression) during the isolation period, changes in sexual desire and reasons, forms of sexual activity, sexual knowledge, etc. I In the last part, we used the Chinese version of the Female Sexual Function Inventory (FSFI) to assess the status of women’s sexual function during this period^39,40^. The FSFI assesses sexual dysfunction in women using six separate titles: cravings, arousal, lubrication, orgasm, sexual success, and pain. A higher score indicates better function.

### Statistical analysis

SPSS 23.0 software was used for data analysis. Means and standard deviations are used to describe continuous variables; counts and proportions are used to describe categorical variables. Student’s *t* test was used for continuous variables, and the chi-square test was used for categorical variables. Logistic regression analysis was used for factor analysis. *P* < 0.05 was regarded as a statistically significant difference.

## Data Availability

The data used to support the findings of this study are included within the article.

## References

[1] Culha MG, Demir O, Sahin O, Altunrende F (2021). Sexual attitudes of healthcare professionals during the COVID-19 outbreak. Int. J. Impot. Res..

[2] Thomas H, Neal-Perry G, Hess R (2018). Female sexual function at midlife and beyond. Obstet. Gynecol. Clinics.

[3] Latif E, Diamond M (2013). Arriving at the diagnosis of female sexual dysfunction. Fertil. Steril..

[4] Pérez-López F (2020). Association of female genital mutilation and female sexual dysfunction: a systematic review and meta-analysis. Eur. J. Obstet. Gynecol. Reprod. Biol..

[5] Biglu M (2016). Effect of female genital mutilation/cutting on sexual functions. Sex. Reprod. Healthc..

[6] Luo H (2017). Elevated free triiodothyronine may lead to female sexual dysfunction in Chinese urban women: a hospital-based survey. Sci. Rep..

[7] Fuchs A (2020). The impact of COVID-19 on female sexual health. Int. J. Environ. Res. Public Health.

[8] Schulman J, Erickson-Schroth L (2019). Mental health in sexual minority and transgender women. Med. Clin. N. Am..

[9] Chew PY (2021). The association between female sexual dysfunction and sexual dysfunction in the male partner: a systematic review and meta-analysis. J. Sex. Med..

[10] Cito G (2021). The impact of the COVID-19 quarantine on sexual life in Italy. Urology.

[11] Kaya Y, Kaya C, Tahta T, Kartal T, Tokgoz VY (2021). Examination of the effect of COVID-19 on sexual dysfunction in women. Int. J. Clin. Pract..

[12] Ibarra FP (2020). Impact of the COVID-19 pandemic on the sexual behavior of the population. The vision of the east and the west. Int. Braz. J. Urol..

[13] Luo YF, Shen HY, Yang SC, Chen LC (2021). The relationships among anxiety, subjective well-being, media consumption, and safety-seeking behaviors during the COVID-19 epidemic. Int. J. Environ. Res. Public Health.

[14] Verbeek M, Hayward L (2019). Pelvic floor dysfunction and its effect on quality of sexual life. Sex. Med. Rev..

[15] Zhang C (2017). A population-based epidemiologic study of female sexual dysfunction risk in Mainland China: prevalence and predictors. J. Sex. Med..

[16] Schiavi M (2020). Love in the time of COVID-19: sexual function and quality of life analysis during the social distancing measures in a group of Italian reproductive-age women. J. Sex. Med..

[17] McCool-Myers M, Theurich M, Zuelke A, Knuettel H, Apfelbacher C (2018). Predictors of female sexual dysfunction: a systematic review and qualitative analysis through gender inequality paradigms. BMC Women’s Health.

[18] Gregory A (2021). Understanding female sexual dysfunction, its causes and treatments. Br. J. Nurs..

[19] Bortolami A, Vanti C, Banchelli F, Guccione A, Pillastrini P (2015). Relationship between female pelvic floor dysfunction and sexual dysfunction: an observational study. J. Sex. Med..

[20] Leeman L, Rogers R (2012). Sex after childbirth: postpartum sexual function. Obstet. Gynecol..

[21] Scavello I, Maseroli E, Di Stasi V, Vignozzi L (2019). Sexual health in menopause. Medicina (Kaunas).

[22] Prime H, Wade M, Browne D (2020). Risk and resilience in family well-being during the COVID-19 pandemic. Am. Psychol..

[23] Pišot S (2022). The differences of Slovenian and Italian daily practices experienced in the first wave of covid-19 pandemic. BMC Public Health.

[24] Clayton A, Juarez E (2019). Female sexual dysfunction. Med. Clin. N. Am..

[25] Deng J (2021). The prevalence of depression, anxiety, and sleep disturbances in COVID-19 patients: a meta-analysis. Ann. N. Y. Acad. Sci..

[26] Li W, Li G, Xin C, Wang Y, Yang S (2020). Challenges in the practice of sexual medicine in the time of COVID-19 in China. J. Sex. Med..

[27] Aitken RJ (2021). COVID-19 and human spermatozoa-Potential risks for infertility and sexual transmission?. Andrology.

[28] Liu S, Han J, Xiao D, Ma C, Chen B (2010). A report on the reproductive health of women after the massive 2008 Wenchuan earthquake. Int. J. Gynaecol. Obstet..

[29] Atarodi-Kashani Z, Kariman N, Ebadi A, Majd H, Beladi-Moghadam N (2017). Sexual function and related factors in Iranian woman with epilepsy. Seizure.

[30] Kaestle C, Allen K (2011). The role of masturbation in healthy sexual development: perceptions of young adults. Arch. Sex. Behav..

[31] Rosenthal C, Thompson L (2020). Child abuse awareness month during the Coronavirus disease 2019 pandemic. JAMA Pediatr..

[32] Gerressu M, Mercer CH, Graham CA, Wellings K, Johnson AM (2008). Prevalence of masturbation and associated factors in a British national probability survey. Arch. Sex. Behav..

[33] Rowland D, Kolba T, McNabney S, Uribe D, Hevesi K (2020). Why and how women masturbate, and the relationship to orgasmic response. J. Sex Marital Ther..

[34] Regnerus M, Price J, Gordon D (2017). Masturbation and partnered sex: substitutes or complements?. Arch. Sex. Behav..

[35] Rowland DL, Hevesi K, Conway GR, Kolba TN (2020). Relationship between masturbation and partnered sex in women: does the former facilitate, inhibit, or not affect the latter?. J. Sex. Med..

[36] Yuksel B, Ozgor F (2020). Effect of the COVID-19 pandemic on female sexual behavior. Int. J. Gynaecol. Obstet..

[37] McCool-Myers M, Theurich M, Zuelke A, Knuettel H, Apfelbacher C (2018). Predictors of female sexual dysfunction: a systematic review and qualitative analysis through gender inequality paradigms. BMC Womens Health.

[38] Brotto L (2021). A randomized trial comparing group mindfulness-based cognitive therapy with group supportive sex education and therapy for the treatment of female sexual interest/arousal disorder. J. Consult. Clin. Psychol..

[39] Wiegel M, Meston C, Rosen R (2005). The female sexual function index (FSFI): cross-validation and development of clinical cutoff scores. J. Sex Marital Ther..

[40] Liu H (2016). Sexual function in cervical cancer patients: psychometric properties and performance of a Chinese version of the Female Sexual Function Index. Eur. J. Oncol. Nurs..

